# Case Report: Life-Threatening Post-operative Hemorrhage in Klippel–Trenaunay Syndrome Associated With Hypofibrinogenemia

**DOI:** 10.3389/fmed.2021.669793

**Published:** 2021-08-09

**Authors:** Hongna Yang, Binfeng Du, Han Liu, Yong Yao, Chen Li

**Affiliations:** ^1^Department of Critical-Care Medicine, Shandong Provincial Hospital Affiliated to Shandong First Medical University, Jinan, China; ^2^Department of Critical-Care Medicine, Qilu Hospital of Shandong University, Shandong University, Jinan, China

**Keywords:** conservative therapy, fibrinogen concentrate, hypofibrinogenemia, klipple-trenaunay syndrome, post-operative hemorrhage, prophylactic transfusion of fibrinogen

## Abstract

Klippel–Trenaunay Syndrome (KTS) is a rare congenital disorder, characterized by venous and lymphatic malformations of the skin, soft tissue, and bone, causing limb hypertrophy. Although, a ruptured hemorrhagic corpus luteum is a rare condition in women of reproductive age, it can result in lethal outcomes. Here, we have described a patient with KTS and hypofibrinogenemia who went through recurrent lethal postoperative bleeding due to a ruptured hemorrhagic corpus luteum. This case suggested that conservative therapy might be the first choice and effective therapy for the patients with KTS, who suffered from bleeding complications of surgical therapy.

## Introduction

Klippel–Trenaunay syndrome (KTS) is an uncommon congenital malformation syndrome involving blood and lymph vessels and disturbed growth of bone and soft tissues (1, 2), which might be associated with the somatic mutations of the PIK3CA (phosphoinositide 3-kinases) gene, but it is not necessary to perform imaging or laboratory/genetic testing to diagnose KTS. The clinical presentations were extremely variable, including cutaneous capillary malformations, lymphatic malformations involving dermal and subcutaneous tissues, varicose veins, and hypertrophy of bone and soft tissue (3). The complications included hematuria, gastrointestinal bleeding, deep vein thrombosis, thrombophlebitis, and pulmonary embolism (4). Venous thromboembolism is the most common complication due to low-flow vascular malformation of KTS (5). Although, there were case reports on hemorrhagic complications of KTS, this bleeding often occurred to the visceral organs affected by venous malformations, such as skin lesions (6), gastrointestinal tract (7), and genitourinary system (8). Although, a nationwide cross-sectional study demonstrated that women with KTS also suffered from hemorrhage complications, the hemorrhagic complication was mainly postpartum bleeding at special periods, such as in pregnancy and in early postpartum (9). However, in the current manuscript, we described a case of a young female patient with KTS and hypofibrinogenemia who suffered from life-threatening post-operative bleeding after explorative laparotomy and excision of ruptured hemorrhagic corpus luteum. More importantly, the female patient was neither pregnant nor in her early postpartum.

## Case Presentation

A 36-year-old female patient with many medical histories was admitted to our department and reported progressively decreased hemoglobin for 6 days duration after explorative laparotomy and excision of ruptured hemorrhagic corpus luteum even after receiving a transfusion of erythrocyte suspension. Her medical histories included a bleeding tendency, poor wound healing, easy bruising, lateral extensive varicose veins, and hypertrophy of the left lower extremity since her early infancy. However, her parents denied a similar medical history or manifestations in the patient. She also reported that she suffered from postpartum bleeding after a cesarean section 6 years ago and survived *via* conservative therapy, including transfusion with erythrocyte suspension (ES) and fresh frozen plasma (FFP). In addition, she was informed to suffer from hypofibrinogenemia and splenomegaly 4 years ago. The level of serum fibrinogen was reported to be maintained at about 1 g/L (normal range: 2–4 g/L). She denied the medical histories of epistaxis, hemarthrosis, and menorrhagia. None of her family members have similar medical histories.

**Figure 1:**
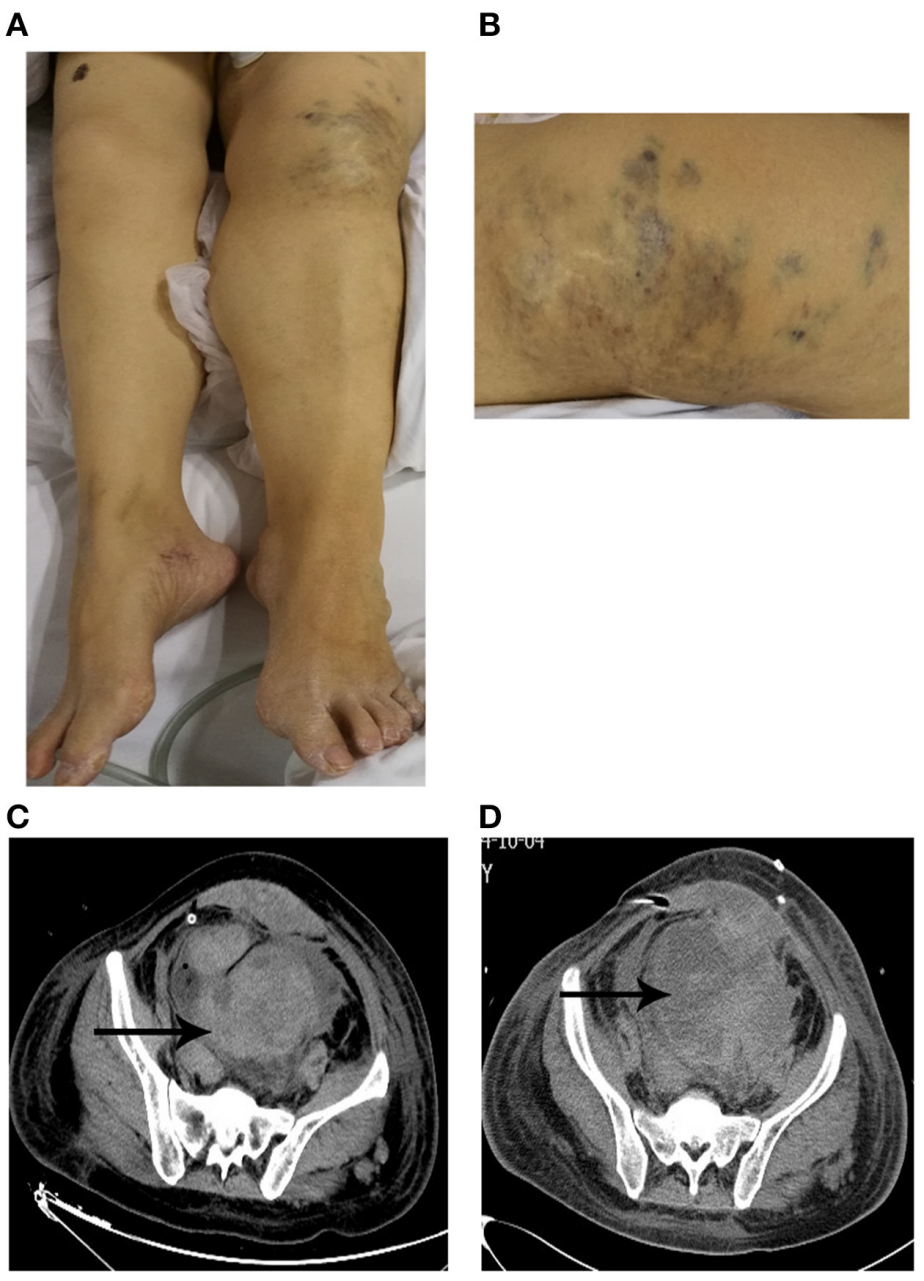
**(A,B)** Patient with Klippel–Trenaunay syndrome. **(A)** The left leg was larger in circumference and longer in length than the right leg. **(B)** Capillary malformation in the left leg. **(C,D)** Unenhanced computer tomography (CT) showed peritoneal diffuse effusion in pelvis with different mixed attenuation values at the admission to our department **(C)** and post-operative period **(D)**.

Physical examination on her admission to our department revealed pallor, tachycardia, varicosities in the left leg (Figure 1A), and hypertrophy of her left leg (Figure 1B). Pulmonary examination was unremarkable. The signs of peritoneal irritation were not detected, and the temperature was 36.4°C. There was red fluid from the abdominal drain. Laboratory tests demonstrated a hemoglobin concentration (HGB) of 68 g/L (115–150 g/L), platelet count (PLT) of 98 ^*^ 10^9^/L (125–135^*^10^9^/L), prothrombin time (PT) of 19.2 s (11–14.5 s), activated partial thromboplastin time (APTT) of 42.7 s (28–45 s), and fibrinogen of 0.75 g/L (2–4 g/L). She was immediately transfused with 2 g of fibrinogen concentrate. Over the next 2 days, laboratory test data persistently deteriorated, although, she was transfused with 14 g of fibrinogen concentrate, 10 U (units) of erythrocyte suspension (ES), 800 ml of fresh frozen plasma (FFP), and 22 U of cryoprecipitate. HGB, PLT, and fibrinogen dropped to 38 g/L, 9 ^*^ 10^9^/L, and 0.44 g/L separately. PT and APPT were prolonged to 25.6 and 56.6 s, separately. Thrombelastogram (TEG) indicated that she was hypocoagulable with low functional fibrinogen and platelet. In addition, there were large amounts of fresh red fluid (800–1,000 ml) from the abdominal drain every day. Abdominal computer tomography (CT) showed a massive hematoma with a diameter of 12.4 ^*^ 8.4 cm (Figure 1C) in her pelvic cavity. However, her vital signs were stable except for tachycardia. Thus, we performed again explorative laparotomy to observe the surgical wound surface. Intraoperatively, extensive oozing of blood from the surgical wound surface was detected, and the massive intra-abdominal hematoma was removed. Intraoperatively, 6 L of blood was evacuated and 6 U of ES, 2 U of PLT, 10 U of cryoprecipitate, and 400 ml of FFP were transfused. However, the post-operative period was still eventful. Post-operatively, the levels of HGB and fibrinogen were maintaining the low levels even with the transfusion of large amounts of ES, FFP, fibrinogen concentrate, and cryoprecipitate, and every day, about 600–1,000 ml of red fluids are drained from the abdominal drain. In addition, abdominal CT (Figure 1D) showed a massive hematoma with a diameter of 11 ^*^ 8.9 cm in the patient's pelvic cavity on day 5 after the operation. The characters of laboratory tests data are demonstrated in Table 1. We performed TEG analysis on days 3 and 10 after the operation, which indicated that she was still hypocoagulable with low functional fibrinogen and platelet. In addition, the whole gene mutation analysis for KTS and congenital hypofibrinogenemia was tested. However, no gene mutation was detected. On day 14 after the operation, TEG analysis was normal with normal functional fibrinogens and platelest. In total, 78 g of fibrinogen concentrate, 23 U of ES, 2 U of PLT, 1,850 ml of FFP, 14.4 g of tranexamic acid, and 78 U of cryoprecipitate were transfused postoperatively until the levels of HGB and fibrinogen were stable. Fortunately, during the process of the disease, the temperature of the patient was maintained at a normal level, and no signs of peritoneal irritation were detected. She was discharged after 35 days of hospitalization with oral contraceptive pills. She was followed up for 6 months with nothing remarkable.

**Table 1:** The characters of laboratory test data.

**Days of admission**	**HGB (g/L)**	**PLT (10^**9**^/L)**	**PT (s)**	**APTT (s)**	**Fib (g/L)**
1	68	98	19.2	42.7	0.75
2	50	43	21.8	49.7	1.25
3	38	9	25.6	56.6	0.44
4	46	8	16.8	42.8	1.52
5	66	21	16	40.4	1.23
6	59	27	14.3	29	1.04
7	60	38	17.4	44.3	1.04
8	59	48	15.1	29.7	1.23
9	60	64	14.1	47	1.55
10	66	63	13	30	1.08
11	76	69	16.9	39.4	1.28
12	72	64	18.1	43.5	1.56
13	74	76	15.1	29.1	1.41
14	77	92	14.7	28.3	1.44
15	86	104	14.8	28.6	1.89
16	86	111	14.6	28.7	1.64
17	87	133	14.7	32.2	2.04
18	90	145	24.7	29.7	2.17
20	96	127	14	29.8	1.43
22	95	122	14.5	31.8	1.07
24	94	117	14.9	30.9	0.96
25	87	102	14.5	31.6	1.26
27	86	94	13.7	33	1.69
29	84	111	14.1	27.7	1.5
31	79	109	14.6	30.3	1.57
33	86	144	13.6	29.7	1.62
35	82	126	16.2	40.3	1.69

HGB, haemoglobin (normal range: 115–150 g/L); PLT, platelet (normal range: 25–135^*^10^9^/L); PT, prothrombin time (normal range: 11–14.5 s); APTT, activated partial thromboplastin time (normal range: 28–45 s); Fib, fibrinogen (normal range: 2–4 g/L).

## Discussion

Ruptured corpus luteum is a common condition in women of reproductive age. Conservative management is becoming the dominating trend, especially for those hemodynamically stable patients, but surgical intervention is required for patients whose vitals are aggravated or where the size of the mass of hemoperitoneum is increasing despite sustained medical therapy in a reasonable time (10). Considering that the laboratory data on HGB, PLT, PT, APTT, and Fib continuously deteriorated even with sustained conservative therapy, the patient received a second explorative laparotomy in a short time to exclude the possibility of the first surgical therapy. However, the postoperative period was eventful. As illustrated in Table 1, these laboratory data were still unstable even if she was receiving a second explorative laparotomy and sustained conservative therapy. In addition, a second massive hematoma formation in a short time and TEG analysis after the second operation (Figure 1C) further questioned the necessity of the surgical therapy.

The diagnosis of KTS is based on two clinical features, which include congenital vascular malformations (capillary malformation, varicosities, or/and lymphatic malformation) and disturbed growth of bone or soft tissue in the length or girth (1). At first, we did not realize the existence of KTS because the patient did not provide the medical history of KTS as well as our limited knowledge. The patient was, for the first time, diagnosed to suffer from KTS according to the physical examination, which included capillary malformation (Figure 1B) and hypertrophy of the left leg (Figure 1A). In addition, venous malformations may also be found in the visceral organs, such as the spleen, which caused splenomegaly (2). Thus, the medical history of splenomegaly further supported the diagnosis of KTS.

The diagnosis of congenital hypofibrinogenemia is based on the detection of a proportional reduction of both antigen and functional fibrinogen concentration. The patient has a medical history of hypofibrinogenemia. Although, the function of fibrinogen at an early time was low, TEG at the late time of hospitalization demonstrated the normal functional fibrinogen. The reduction of functional fibrinogen might be secondary to the first surgical trauma. In addition, no gene mutation for congenital fibrinogen deficiency was detectable. Thus, the diagnosis of congenital hypofibrinogenemia needed to be further discussed or questioned.

The complications of KTS included pain, cellulitis, thrombophlebitis, deep vein thrombosis, arthritis, bleeding, and consumptive coagulopathy (6, 11, 12). Consumptive coagulopathy, known as localized intravascular coagulopathy (LIC), is characterized by reduced fibrinogen level, an elevated D-dimer level, and reduced or normal platelet counts (13, 14). According to the medical history, we concluded that hypofibrinogenemia was the presentation of LIC, which was the complication of KTS. LIC was able to progress into disseminated intravascular coagulopathy (DIC), which was consistent with the first laboratory data before the second operation. More importantly, surgical or radiological interventional procedures were the risk factors in the conversation of LIC into DIC (15). Thus, LIC was able to explain the postoperative bleeding or massive hematoma formation in the pelvic cavity.

It was reported that preoperative replacement therapy with fibrinogen concentrate in a patient with KTS and severe hypofibrinogenemia produced an improvement in abnormal clotting and was enough in decreasing the risks of postoperative bleeding (12). We also observed that the laboratory data were gradually stable in the late postoperative period under sustained conservative treatment, especially after the massive hemoperitoneum formation. Thus, these indicated that sustained conservative therapy was an effective treatment even for a postoperative patient with KTS and hypofibrinogenemia. In addition, contraceptives are recommended for women of reproductive age to prevent future ruptured hemorrhagic corpus luteum by suppression of ovulation. However, oral contraceptive pills increase the risks of thrombotic events. In addition, KTS also results in thrombotic events. Thus, we suggested that the patient pay attention to such side effects after she was discharged.

## Conclusion

Conservative therapy is the first choice and effective therapy for patients with KTS and/or hypofibrinogenemia, who went through a bleeding complication of surgical therapy due to a ruptured hemorrhagic corpus luteum. More importantly, prophylactic transfusion of fibrinogen concentrate might improve or shorten the postoperative progress. In addition, contraceptives are recommended for women of reproductive age with high risks of bleeding due to accompanying diseases to prevent relapse of ruptured hemorrhagic corpus luteum by suppression of ovulation.

## Data Availability Statement

The original contributions presented in the study are included in the article/supplementary material, further, inquiries can be directed to the corresponding author/s.

## Ethics Statement

Written Informed Consent was obtained from the patient for the publication of this case report.

## Author Contributions

HY wrote the manuscript. BD, HL, and YY helped in the collection of the clinical data and secured the consent of the patients. CL helped in the diagnosis. All authors contributed to the article and approved the submitted version.

## Conflict of Interest

The authors declare that the research was conducted in the absence of any commercial or financial relationships that could be construed as a potential conflict of interest.

## Publisher's Note

All claims expressed in this article are solely those of the authors and do not necessarily represent those of their affiliated organizations, or those of the publisher, the editors and the reviewers. Any product that may be evaluated in this article, or claim that may be made by its manufacturer, is not guaranteed or endorsed by the publisher.
